# LncRNA SNHG15 contributes to proliferation, invasion and autophagy in osteosarcoma cells by sponging miR-141

**DOI:** 10.1186/s12929-017-0353-9

**Published:** 2017-07-18

**Authors:** Ke Liu, Yi Hou, Yunke Liu, Jia Zheng

**Affiliations:** Department of Orthopaedics, Henan Provincial People’s Hospital, No. 7 Weiwu Road, Zhengzhou, 450003 China

**Keywords:** lncRNA SNHG15, miR-141, Sponge, Osteosarcoma

## Abstract

**Background:**

LncRNA small nucleolar RNA host gene 15 (SNHG15) was reported to play an oncogenic role in tumors. However, the role of SNHG15 and its molecular mechanism in osteosarcoma (OS) cells are largely unknown.

**Methods:**

qRT-PCR was performed to evaluate the expression levels of SNHG15 and miR-141 in OS tissues and cells. Cell transfection with different siRNAs, miRNAs or pcDNAs into U2OS and MG63 cells were carried out by Lipofectamine 2000. The effects of SNHG15 and miR-141 on OS cell proliferation, invasion and the levels of autophagy-related proteins were analyzed by MTT assay, Transwell invasion/migration assay and western blot, respectively. Luciferase reporter assay was used to confirm whether SNHG15 could directly interact with miR-141.

**Results:**

We found that up-regulation of SNHG15 was inversely correlated with miR-141 expression in OS tissues. SNHG15 knockdown and miR-141 overexpression significantly suppressed cell proliferation, invasion, migration and autophagy while SNHG15 overexpression and miR-141 repression exhibited the opposite effects on OS cells. Besides, SNHG15 could directly interact with miR-141 and regulate its expression. Furthermore, miR-141 suppressing significantly overturned the inhibition on proliferation, invasion, migration and autophagy mediated by SNHG15 knockdown while miR-141 overexpression remarkably attenuated SNHG15 overexpression-induced proliferation, invasion, migration and autophagy in OS cells.

**Conclusion:**

Our data showed that SNHG15 contributes to proliferation, invasion, migration and autophagy in OS by negatively regulating miR-141, providing a new potential target and prognostic biomarker for the treatment of OS.

## Background

Osteosarcoma (OS) is the most frequent primary malignant tumor of the skeleton worldwide that predominately affects the rapid growth of bones in children and adolescents, comprising approximately 15% of all bone malignancies [1]. The 5-year survival rate of patients diagnosed with OS has significantly increased to approximately 60–70% due to the advent of adjuvant and neoadjevant chemotherapy [2]. However, numerous OS patients are still insensitive to chemotherapy and the overall survival rate of OS patients has reached a plateau due to local relapse or distant metastasis even after curative excision of the primary tumor and intensive chemotherapy [3, 4]. Thus, it is urgently needed to develop novel molecular therapeutic targets for OS and better understand the underlying molecular mechanism of OS pathogenesis and progression.

Recently, advances in human genome sequencing have shown that more than 90% of the human genome is extensively transcribed but only approximately 2% of it serves as protein-coding genes [5]. The majority of the remaining transcripts are non-coding RNAs (ncRNAs) with no protein-coding capacity, including small ncRNAs, especially microRNAs (miRNAs), and long non-coding RNAs (lncRNAs) based on their transcript size. miRNAs have been demonstrated to be involved in regulating various pathological processes, such as cellular proliferation, differentiation, migration, as well as apoptosis [6]. It is well documented that miRNAs function as either oncogenes or tumor suppressors via specifically regulating protein-coding genes [7]. In recent years, mounting evidences have indicated that multiple miRNAs have been identified as key epigenetic regulators in development and progression of tumors, including OS [8]. miR-141, belonging to miR-200 family, was found to be down-regulated in OS and contributed to OS tumorigenesis [9, 10]. Overexpression of miR-141 not only inhibited osteosarcoma cell proliferation but also induced cell apoptosis [9]. Mei et al. reported that activating the miR-200 family might have an anti-osteosarcoma effect and miR-200 family might be the targets for osteosarcoma treatment [11]. He et al. reported that miR-141 as a tumor-suppressor and miR-141 overexpression could suppress proliferation but induced apoptosis through down-regulating H19 or miR-675 in osteosarcoma [12]. Besides, miR-141 also served as a tumor suppressor in nasopharyngeal carcinoma [13], hepatocellular carcinoma [14], and gastric cancer [15].

LncRNAs are evolutionarily conserved ncRNAs with more than 200 nucleotides in length [16]. In recent years, many reports have shown that lncRNAs play an oncogenic or tumor suppressive role in various cancers, such as breast, gastric, colorectal cancers and hepatocellular carcinoma [17–19]. Emerging evidences have demonstrated that dysregulation of lncRNAs has been implicated in multiple cellular biological processes, such as cell proliferation, apoptosis, cancer metastasis, as well as tumorigenesis [20, 21]. Furthermore, several lncRNAs were reported to be involved in OS progression and tumorigenesis [22]. For example, Zheng et al. found that lncRNA HOTAIR, one of the well-studied lncRNAs, functioned as a carcinogenic lncRNA, promoting proliferation and inhibiting apoptosis of OS MG-63 cells in vitro [23]. Zhao et al. reported that lncRNA HNF1A-antisense 1 (HNF1A-AS1) was markedly up-regulated and promoted OS progression by regulating the activity of the Wnt/β-catenin pathway [24]. Li et al. showed that lncRNA urothelial carcinoma associated 1 (UCA1) was overexpressed in OS tissues and cells and contributed to OS initiation and progression [25]. Small nucleolar RNA host gene 15 (SNHG15) is located on chromosome 7p13 but does not encode protein. Previous studies reported that overexpression of SNHG15 sensitized human cells to death in response to various environmental stresses, such as cisplatin, cycloheximide, and mercury (II) oxide and has been identified as surrogate indicators for stress response [26, 27]. Up to now, no study reported the clinical role of SNHG15 and its biological functions in OS.

Autophagy is a process in which subcellular membranes undergo dynamic morphological changes that lead to the degradation of cellular proteins and cytoplasmic organelles [28]. Autophagy is an important cellular response to stress or starvation. Many studies have showed that autophagy played a significant role in human cancers including osteosarcoma [29, 30]. Recently, several studies revealed that miRNAs could regulate autophagy in various cancer types. For example, miR-22 could inhibit autophagy in osteosarcoma cells during chemotherapy [31]. miR-200 family microRNAs could regulate autophagy in vitro and in vivo in osteosarcoma [11]. Moreover, miR-141 was reported to regulate autophagy in many human diseases [32, 33]. In our study, we investigated the expressions of SNHG15 and miR-141 and their biological functions in OS, then performed bioinformatics-based target prediction analysis and found that SNHG15 contains one conserved target site of miR-141. Based on these findings, we hypothesize that SNHG15 might regulate proliferation, invasion and autophagy in osteosarcoma cells by sponging miR-141.

## Methods

### Patients and tumor samples

Thirty-five paired cancer tissues and the adjacent normal tissues were collected from primary OS patients underwent resection during initial surgery at the Henan Provincial People’s Hospital between 2014 and 2015. Surgically resected tissues samples were immediately frozen in liquid nitrogen and subsequently stored at −80 °C until use. The resected nodules of OS were histopathologically confirmed. The present study was performed with written informed consent from all participants for the use of their tissues.

### Cell lines and cell culture

The human OS cell lines (143B, U2OS, HOS, MG63, and SaOS2) and osteoblastic cell line HFOB1.19 were all purchased from American Type Culture Collection (ATCC, Manassas, VA, USA). These OS cell lines were cultured in Dulbecco’s modified Eagle’s medium (DMEM; Gibco, Grand Island, NY, USA) containing 10% fetal bovine serum (FBS; Invitrogen, Carlsbad, CA, USA), as well as 100 U/mL penicillin and 100 μg/mL streptomycin in a humidified chamber with 5% CO_2_ at 37 °C. HFOB1.19 cells were maintained in DMEM/Ham’s F-12 (1:1) medium supplemented with 10% FBS and 0.3 mg/mL G418 (Gibco) under the same conditions.

### Cell transfection

The siRNAs specifically targeting SNHG15 (si-SNHG15–1, si-SNHG15–2 and si-SNHG15–3), scrambled negative control (si-control), pcDNA-SNHG15, empty pcDNA vector (vector), miR-141 mimics (miR-141), mimics negative control (miR-control), anti-miR-141 and anti-miR-control were commercially synthesized by Genepharma (Shanghai, China). For transient transfection, U2OS and MG63 cells were plated into six-well plates (2 × 10^5^/well) and routinely maintained for 24 h at 37 °C. Then the cells were transfected with siRNAs, pcDNA-SNHG15, vector, miR-141, miR-control, anti-miR-141, anti-miR-control, si-SNHG15 + anti-miR-141, si-SNHG15 + anti-miR-control, pcDNA-SNHG15 + miR-141, or pcDNA-SNHG15 + miR-control by Lipofectamine 2000 (Invitrogen) according to the manufacturer’s protocol. Subsequent experiments were performed at 48 h post transfection. The sequences of si-RNAs, anti-miR-141 are as follows: si-SNHG15–1 (Sense: 5′-CAG GTA GAC CGT GCA CGT AA-3′, Anti-sence:3′-CCT TGA TGC GTT GCC AGC AGA-5′), si-SNHG15–2 (Sense: 5′-CCG TGC GTA AAC GTT TGC CA-3′, Anti-sence: 3′-TGG CGG TAA CGT AAA TGC G-5′), si-SNHG15–3 (Sense: 5′-ACG GTG GCA ACG TGC GTG GCC A-3′, Anti-sence: 3′-GCC TGC AAC GGT GCA AAT GCG-5′), anti-miR-141 (CCA UCU UUA CCA GAC AGU GU UA).

### Quantitative real-time PCR

Total RNA was extracted from tissues specimens and cultured cells using Trizol reagent (Invitrogen). Total RNA concentration and purity were detected by the ratio of absorbance at 260/280 nm using a NanoDrop ND-1000 spectrophotometer (Thermo Fisher Scientific, Waltham, MA, USA). Total RNA (2 μg) was reversely transcribed into cDNA using the Reverse Transcription System Kit (Takara; Dalian, China). The expression levels of SNHG15 and miR-141 were determined by RT-PCR using SYBR Premix ExTaq II kit (Takara) and mirVanaTM qRT-PCR miRNA Detection Kit (Ambion, Austin, TX, USA) on an ABI 7500 PRISM 7500 Sequence Detection System (Applied Biosystems, Foster City, CA, USA), respectively. The relative expression levels of SNHG15 and miR-141 were calculated by the 2^-ΔΔCt^ method and normalized to GAPDH expression and the relative expression levels of miR-141 were normalized to *U6*. PCR conditions for SNHG15 were as follows: 2 min at 50 °C, 10 min at 95 °C, 45 cycles of 95 °C for 15 s, 60 °C for 30 s, 72 °C for 45 s; PCR conditions for miR-141 was 10 min at 95 °C, 45 cycles of 95 °C for 10 s, 60 °C for 20 s and 72 °C for 12 s. Each experiment was performed at least three times. The primer sequences used are as follows: SNHG15 (Sense: 5′-CAA CCA TAG CGG TGC AAC TGT GC-3′, Anti-sence: 3′-GGC TGA ACC AAG TTG CAA GTC ATG-5′); miR-141 (Sense: 5′-GGG CAT CTT CCA GTA CAG T-3′, Anti-sence: 3′-CAG TGC GTG TCG TGG AGT-5′); GAPDH (Sense: 5′-CAG TGC CAG CCT CGT CTA T-3′, Anti-sence: 3′-AGG GGC CAT CCA CAG TCT TC-5′); U6 (Sense: 5′-CTCGCTTCGGCAGCACATATACT-3′, Anti-sence: 3′-ACG CTT CAC GAA TTT GCG TGT C-5′).

### MTT assay

Cell proliferation ability was evaluated by MTT assay. Briefly, transfected U2OS and MG63 cells were seeded into 96-well plates at a density of 5 × 10^3^/well and routinely cultured for 24 h, 48 h, 72 h and 96 h, respectively. At the indicated time, 20 μL MTT regents (5 mg/mL; Sigma-Aldrich, Irvine, Ayrshire, UK) was supplemented into each well at a final MTT concentration of 0.45 mg⁄mL and incubated for another 4 h at 37 °C in a humidified chamber. Subsequently, the medium was removed and 150 μL of dimethylsulfoxide (DMSO; Sigma-Aldrich) was added to each well to dissolve the blue formazan crystals for 30 min. The absorbance value at 490 nm was recorded on the Model 680 microplate reader (Bio-Rad, Hercules, CA, USA). The measurements for each sample were performed in triplicate.

### Transwell invasion and migration assays

For the invasion and migration assays, 48 h after transfection, approximately 2 × 10^5^ U2OS or MG63 cells in 200 μL serum-free media were transferred to the upper chamber (8.0 μm; Costar, Corning, NY, USA) with a porous membrane pre-coated with (invasion) or without (migration) Matrigel solution (BD, Franklin Lakes, NJ, USA). Meanwhile, DMEM Medium (500 μL) containing 10% fetal bovine serum was added to the lower chamber as a chemoattractant. After 24 h of incubation at 37 °C, the non-invading or non-migration cells on the upper membrane were removed mechanically. Cells invading or migrating to the lower surface of the chamber were fixed in methanol, stained with 4 g/L crystal violet for 2 h. The numbers of invasive or migration cells in each well were counted from five random fields using a microscope (Olympus Corp., Tokyo, Japan). Experiments were independently performed at least three times.

### Luciferase reporter assay

Online softwares including Diana Tools (http://diana.imis.athena-innovation.gr/), Starbase (http://starbase.sysu.edu.cn/) and TargetScan (http://www.targetscan.org) showed that sequences of SNHG15 and miR-141 have binding domain as showed in Fig. 4a. The mRNA sequence of SNHG15 containing the putative binding sites of miR-141 was separately inserted into the *Kpn*I and *Bgl*II sites of pGL3-Basic (Promega, Madison, WI, USA) to form the luciferase reporter vector pGL3-SNHG15-wild-type (pGL3-SNHG15-WT, the whole SNHG15 cDNA fragment). To mutate the putative binding site of miR-141 in SNHG15 gene, the sequence of putative binding site was replaced as indicated to construct pGL3-SNHG15-mutant (pGL3-SNHG15-MUT). U2OS and MG63 cells (3 × 10^4^) were plated in 24-well plates and cultured for overnight at 37 °C. Subsequently, cells were cotransfected with pGL3-SNHG15-WT or pGL3-SNHG15-MUT (100 ng) and miR-141 or miR-control (50 nM) by Lipofectamine 2000 (Invitrogen). Each sample was cotransfected with the pGL3-TK plasmid (Promega) containing Renilla luciferase gene to monitor transfection efficiency. After 24 h of cotransfection, the cells were collected for analyzing the luciferase activities by a Dual-Luciferase Reporter Assay System (Promega). The relative luciferase activity of each sample was normalized to Renilla luciferase activity. The assays were independently performed three times.

### Western blot analysis

Transfected U2OS and MG63 cells were harvested and lysed using a lysis buffer (Beyotime, Shanghai, China) with protease inhibitor (Sigma-Aldrich). Following incubation on ice for 30 min, cell lysates were centrifugated at 12,000 rpm for 5 min. Total protein concentrations in different samples were measured by a BCA protein assay kit (Beyotime). Equal amounts of protein (20 μg) were denatured after heating in boiling water for 10 min and separated on a 12% sodium dodecyl sulfate polyacrylamide gel electrophoresis (SDS-PAGE). Then proteins were transferred onto polyvinylidene fluoride (PVDF) (Millipore, Billerica, MA, USA) at 100 V for 2 h at 4 °C and subsequently blocked in 5% non-fat milk for 1 h at room temperature. Membranes were immunoblotted with the indicated primary antibodies: anti-Atg5, anti-LC3-I, anti-LC3-II, anti-p62, and anti-β-actin (Cell Signaling Technology, Danvers, MA, USA) overnight at 4 °C. After washed three times with TBST, the membranes were incubated with horseradish peroxidase-conjugated secondary antibody anti-IgG for 1 h at room temperature. Finally, the proteins were visualized using an enhanced chemiluminescence detection system (Millipore) and quantified by densitometry using Quantity One software. β-actin was used for the internal control.

### Statistical analysis

All data were presented as the mean ± standard error (S.E.M.) from at least 3 independent experiments. Statistical significances were assessed by two-tailed Student’s *t*-test or one-way multivariate analysis of variance (ANOVA) in SPSS 19.0 statistical software (SPSS, Inc., Chicago, IL, USA). *P* value less than 0.05 was considered statistically significant.

## Results

### SNHG15 was negatively correlated with miR-141 expression in OS tissues

**Fig. 1:**
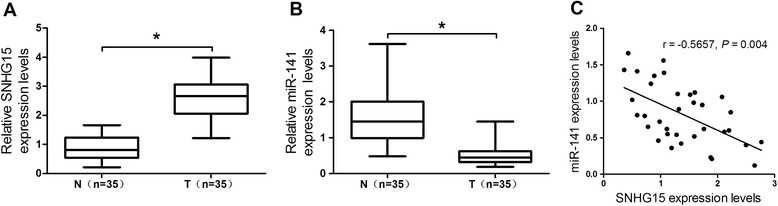
Expression levels of SNHG15 and miR-141 in OS tissues. qRT-PCR was performed to evaluate the expression levels of SNHG15 (**a**) and miR-141 (**b**) in 35 paired OS tissues and the adjacent normal tissues. GAPDH was used as the endogenous control. (**c**) Correlation between SNHG15 and miR-141 expression. *P < 0.05 vs. control group

To define the roles of SNHG15 and miR-141 in OS progression, we first examined the expression levels of SNHG15 and miR-141 in 35 paired OS tissues and the adjacent normal tissues by qRT-PCR. As presented in Fig. 1a and b, SNHG15 expression was significantly higher and miR-141 expression was dramatically lower in 35 paired OS tissues than that in adjacent normal tissues. Interestingly, by comparing the relationship of expression levels between SNHG15 and miR-141, we observed that SNHG15 was negatively correlated with miR-141 expression in OS tissues (*r*  =  −0.5657, *P* = 0.004; Fig. 1c). These data indicated that SNHG15 and miR-141 may be involved in the progression and prognosis of OS.

### SNHG15 promoted OS cell proliferation, invasion, migration and autophagy

**Fig. 2:**
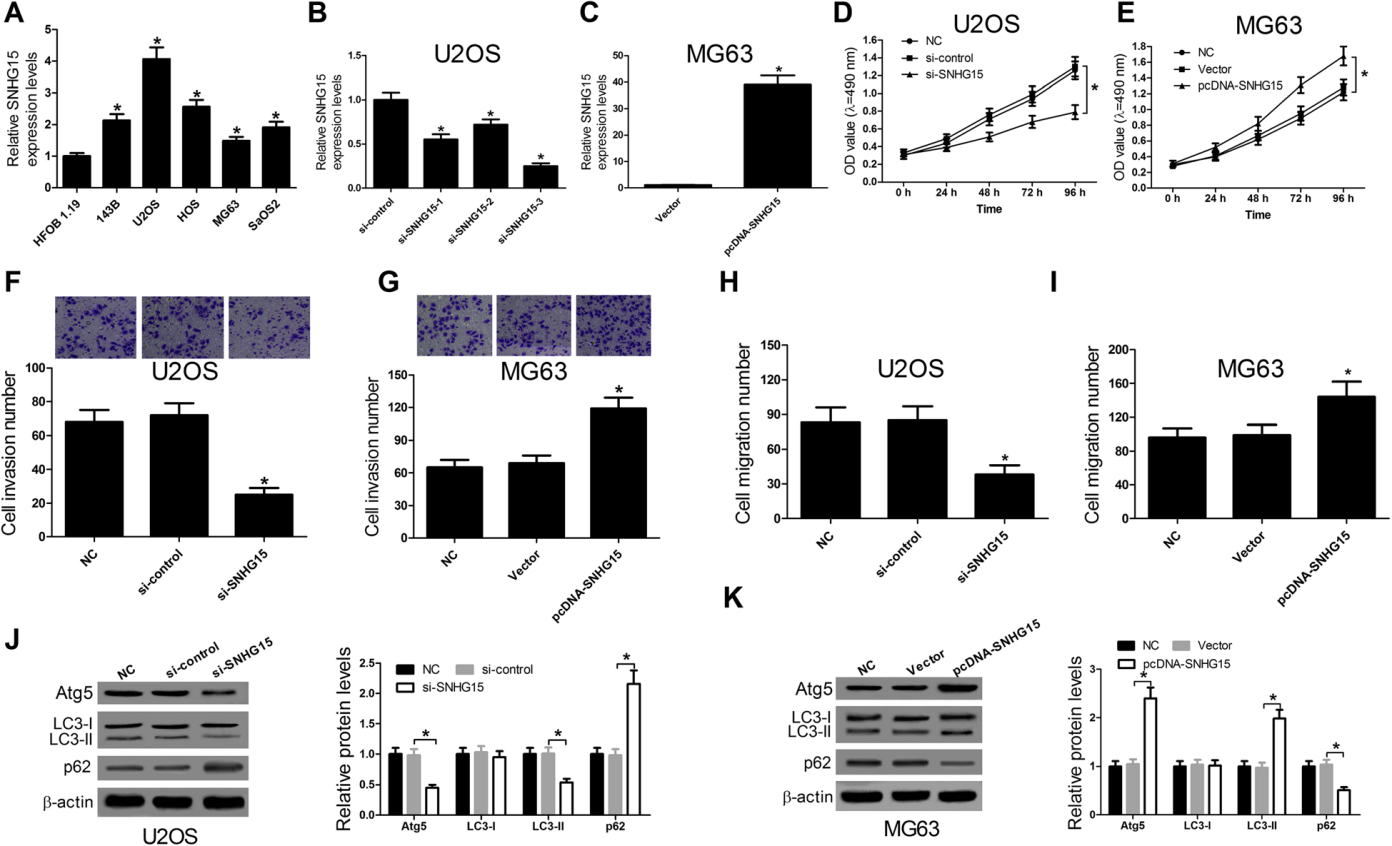
Effects of SNHG15 on OS cell proliferation, invasion, migration and autophagy. **a** qRT-PCR was performed to determine the expressions of SNHG15 in five OS cell lines (143B, U2OS, HOS, MG63 and SaOS2) and osteoblastic cell line HFOB1.19. GAPDH was used as the endogenous control. **b** The expression of SNHG15 in U2OS cells transfected with si-SNHG15–1, si-SNHG15–2, si-SNHG15–3 or si-control was evaluated by qRT-PCR. GAPDH was used as the endogenous control. **c** The expression of SNHG15 in MG63 cells transfected with pcDNA-SNHG15 or vector was assessed by qRT-PCR. GAPDH was used as the endogenous control. MTT assay was carried out to detect cell proliferation at 24 h, 48 h, 72 h, and 96 h in U2OS cells (**d**) transfected with si-SNHG15 or si-control and MG63 cells (**e**) transfected with pcDNA-SNHG15 or vector. Transwell invasion assay in U2OS cells (**f**) transfected with si-SNHG15 or si-control and MG63 cells (**g**) transfected with pcDNA-SNHG15 or vector were performed to detect cell invasiveness. Transwell migration assay in U2OS cells (**h**) transfected with si-SNHG15 or si-control and MG63 cells (**i**) transfected with pcDNA-SNHG15 or vector were performed to detect cell migration. Western blot was used to evaluate the levels of Atg5, LC3-I, LC3-II and p62 in U2OS cells (**j**) transfected with si-SNHG15 or si-control and MG63 cells (**k**) transfected with pcDNA-SNHG15 or vector. β-actin was used as the internal control. *P < 0.05 vs. control group

A further qRT-PCR analysis of SNHG15 expression in OS cells showed that aberrantly elevated expression of SNHG15 was observed in all five OS cell lines (143B, U2OS, HOS, MG63 and SaOS2) compared with osteoblastic cell line HFOB1.19 (Fig. 2a). To explore the biological functions of SNHG15 on OS progression, we knocked down SNHG15 expression in U2OS cells by transfection of si-SNHG15 and enhanced SNHG15 expression in MG63 cells by transfection of pcDNA-SNHG15. As compared with si-control, the efficiency of si-SNHG15 knockdown by si-SNHG15–1, si-SNHG15–2 and si-SNHG15–3 was obtained approximately 45%, 28% and 75% in U2OS cells, respectively (Fig. 2b). Thus, si-SNHG15–3 was chosen for the following experiments. In addition, the expression of SNHG15 was significantly enhanced in MG63 cells transfected with pcDNA-SNHG15 in comparison with cells transfected with vectors (Fig. 2c). MTT assay results disclosed that SNHG15 knockdown remarkably inhibited cell proliferation at 48 h, 72 h, and 96 h in U2OS cells compared with si-control transfected cells (Fig. 2d), whereas elevated expression of SNHG15 markedly promoted cell proliferation at 72 h and 96 h in MG63 cells compared with cells transfected with vectors (Fig. 2e). To further explore the effects of SNHG15 on cell invasion, Transwell invasion assay and Transwell migration assay were performed. As shown in Fig. 2f and g, the number of invasive cells was strikingly reduced in si-SNHG15 transfected U2OS cells compared with si-control group while the number of invasive cells was obviously improved in pcRNA-SNHG15 transfected MG63 cells compared with vector group. As shown in Fig. 2h and i, the number of migration cells was strikingly reduced in si-SNHG15 transfected U2OS cells compared with si-control group while the number of migration cells was obviously improved in pcRNA-SNHG15 transfected MG63 cells compared with vector group. Furthermore, to investigate the effects of SNHG15 on autophagy levels of OS cells, the levels of autophagy-related proteins Atg5 (related to the autophagosomes formation), LC3-I (cytosolic form of key protein LC3 in autophagosome formation), LC3-II (active membrane-bound form of LC3) and p62 (SQSTM1) were assessed by western blot. The levels of LC3-II have been shown to be a reliable indicator of autophagy, and the ubiquitin-binding protein p62 is an autophagy substrate, which is efficiently degraded by autophagy. The degradation of p62 means that autophagy levels are enhanced. The western blot results indicated that the levels of Atg5 and LC3-II and the ratio of LC3-II/ LC3-I were both significantly decreased in si-SNHG15 transfected U2SO cells, meanwhile, the levels of p62 were increased (Fig. 2j) compared with si-control transfected cells, suggesting that SNHG15 knockdown inhibited autophagy of OS cells. Besides, the levels of Atg5, LC3-II and the ratio of LC3-II/ LC3-I were conspicuously increased but the levels of p62 were decreased in pcDNA-SNHG15 transfected MG63 cells compared with cells transfected with vector (Fig. 2k), indicating that SNHG15 overexpression enhanced autophagy of OS cells. Taken together, these results suggested that SNHG15 may play an oncogenic role in regulating OS biological progress including proliferation, invasion, migration, and autophagy.

### miR-141 inhibited OS cell proliferation, invasion and autophagy

**Fig. 3:**
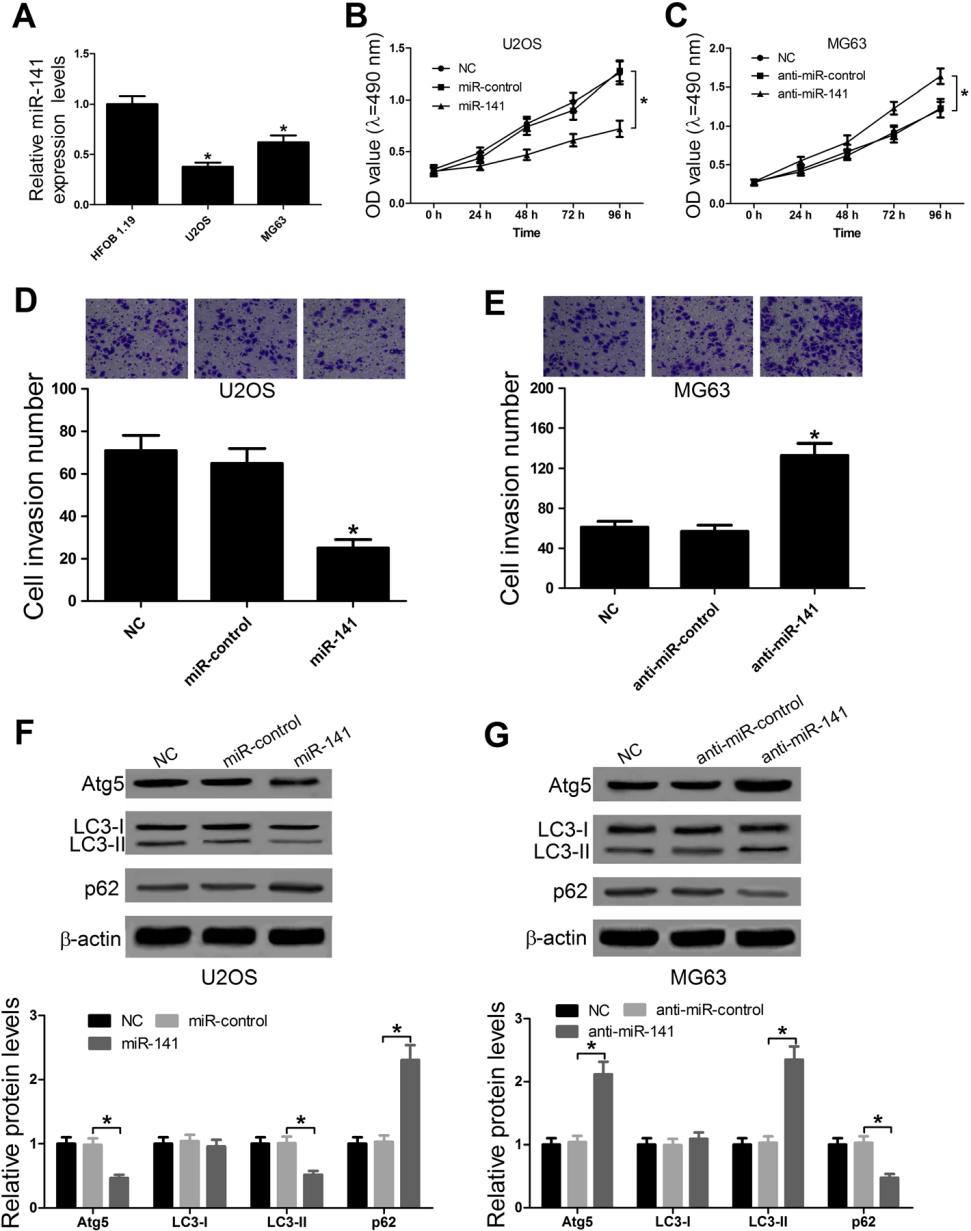
Effect of miR-141 on OS cell proliferation, invasion and autophagy. **a** The expressions of miR-141 in OS cell lines (U2OS and MG63) and osteoblastic cell line HFOB1.19 were analyzed by qRT-PCR. GAPDH was used as the endogenous control. Cell proliferation in U2OS cells (**b**) transfected with miR-141 or miR-control and MG63 cells (**c**) transfected with anti-miR-141 or anti-miR-control at 24 h, 48 h, 72 h and 92 h was examined by MTT assay. The number of invasive cells in U2OS cells (**d**) transfected with miR-141 or miR-control and MG63 cells (**e**) transfected with anti-miR-141 or anti-miR-control were determined by Transwell invasion assay. The levels of Atg5, LC3-I, LC3-II and p62 in U2OS cells (**f**) transfected with miR-141 or miR-control and MG63 cells (**g**) transfected with anti-miR-141 or anti-miR-control were detected by western blot. β-actin was used as the internal control. *P < 0.05 vs. control group

Previous studies have demonstrated that miR-141 was down-regulated in OS and acted as a tumor suppressor in OS cells [9]. To further confirm the role of miR-141 in OS cells, the expression of miR-141 in OS cells was first detected by qRT-PCR and the results exhibited that a lower expression of miR-141 was observed in U2OS and MG63 cells than that in HFOB1.19 cells (Fig. 3a). As expected, MTT assay suggested that forced expression of miR-141 by miR-141 mimics markedly inhibited cell proliferation at 48 h, 72 h and 96 h in U2OS cells compared with si-control transfected cells (Fig. 3b), whereas reduced expression of miR-141 by anti-miR-141 significantly promoted cell proliferation at 72 h and 96 h in MG63 cells compared with anti-miR-control transfected cells (Fig. 3c). Besides, Transwell invasion assay suggested that miR-141 overexpression dramatically reduced the number of invasive cells in U2OS cells compared with cells transfected with si-control (Fig. 3d) while miR-141 down-regulation obviously increased the number of invasive cells in MG63 cells in comparison with cells transfected with anti-miR-control (Fig. 3e). Furthermore, miR-141 overexpression significantly decreased the levels of of Atg5, LC3-II and the ratio of LC3-II/ LC3-I and increased the levels of p62 in U2OS cells compared with miR-control group (Fig. 3f) while miR-141 down-regulation dramatically increased the levels of Atg5, LC3-II and the ratio of LC3-II/ LC3-I and decreased the levels of p62 in MG63 cells compared with anti-miR-control group (Fig. 3g). Therefore, these results verified that miR-141 functioned as a tumor suppressor in OS cells.

### SNHG15 could directly interact with miR-141 and regulate its expression

**Fig. 4:**
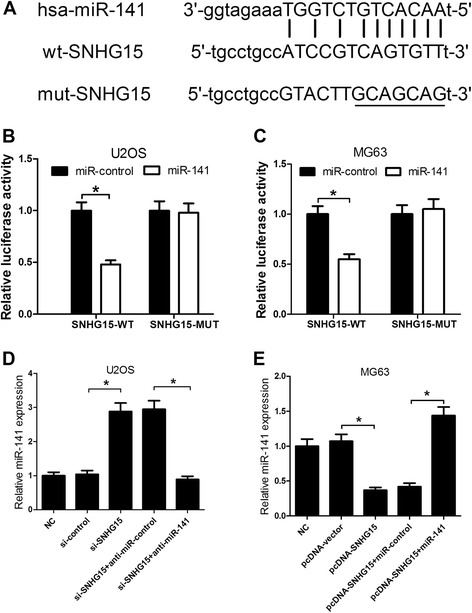
The regulatory relationship between SNHG15 and miR-141. **a** The bioinformatics predicted binding sites of miR-141 on SNHG15. Luciferase reporter assay was performed in U2OS (**b**) and MG63 (**c**) cells cotransfected with pGL3-SNHG15-WT or pGL3-SNHG15-MUT and miR-141 or miR-control. **d** qRT-PCR was carried out to determine the expressions of miR-141 in U2OS cells transfected with si-SNHG15, si-control, si-SNHG15 + anti-miR-141, or si-SNHG15 + anti-miR-control. GAPDH was chosen as the internal control. **e** qRT-PCR was carried out to examine the expression of miR-141 in MG63 transfected with pcDNA-SNHG15, vector, pcDNA-SNHG15 + miR-141, or pcDNA-SNHG15 + miR-control. GAPDH was chosen as the internal control. *P < 0.05 vs. control group

Recently, a large number of evidences have revealed that lncRNAs could serve as miRNA sponges to specifically bind miRNA [34, 35]. Thus, we tried to detect whether SNHG15 could function as a molecular sponge of miR-141. Bioinformatics were used to predict the potential miRNA target sites. Surprisingly, SNHG15 contained the potential binding sites of miR-141 (Fig. 4a). We performed luciferase reporter assay to confirm whether miR-141 could directly bind to SNHG15 and the results demonstrated that the luciferase activity in pGL3-SNHG15-WT cells was remarkably inhibited by miR-141 overexpression in U2OS (Fig. 4b) and MG63 (Fig. 4c) cells, while miR-141 overexpression had no obvious inhibitory effect on pGL3-SNHG15-MUT cells. Furthermore, qRT-PCR results showed that SNHG15 knockdown significantly improved the expression level of miR-141, but anti-miR-141 strikingly attenuated this effect in U2OS cells (Fig. 4d). Additionally, SNHG15 overexpression dramatically reduced miR-141 expression, in contrast, miR-141 overexpression markedly relieved the reduction of miR-141 level in MG63 cells (Fig. 4e). These findings exhibited that SNHG15 may serve as a molecular sponge for miR-141.

### SNHG15 promoted OS cell proliferation, invasion and autophagy in by sponging miR-141

**Fig. 5:**
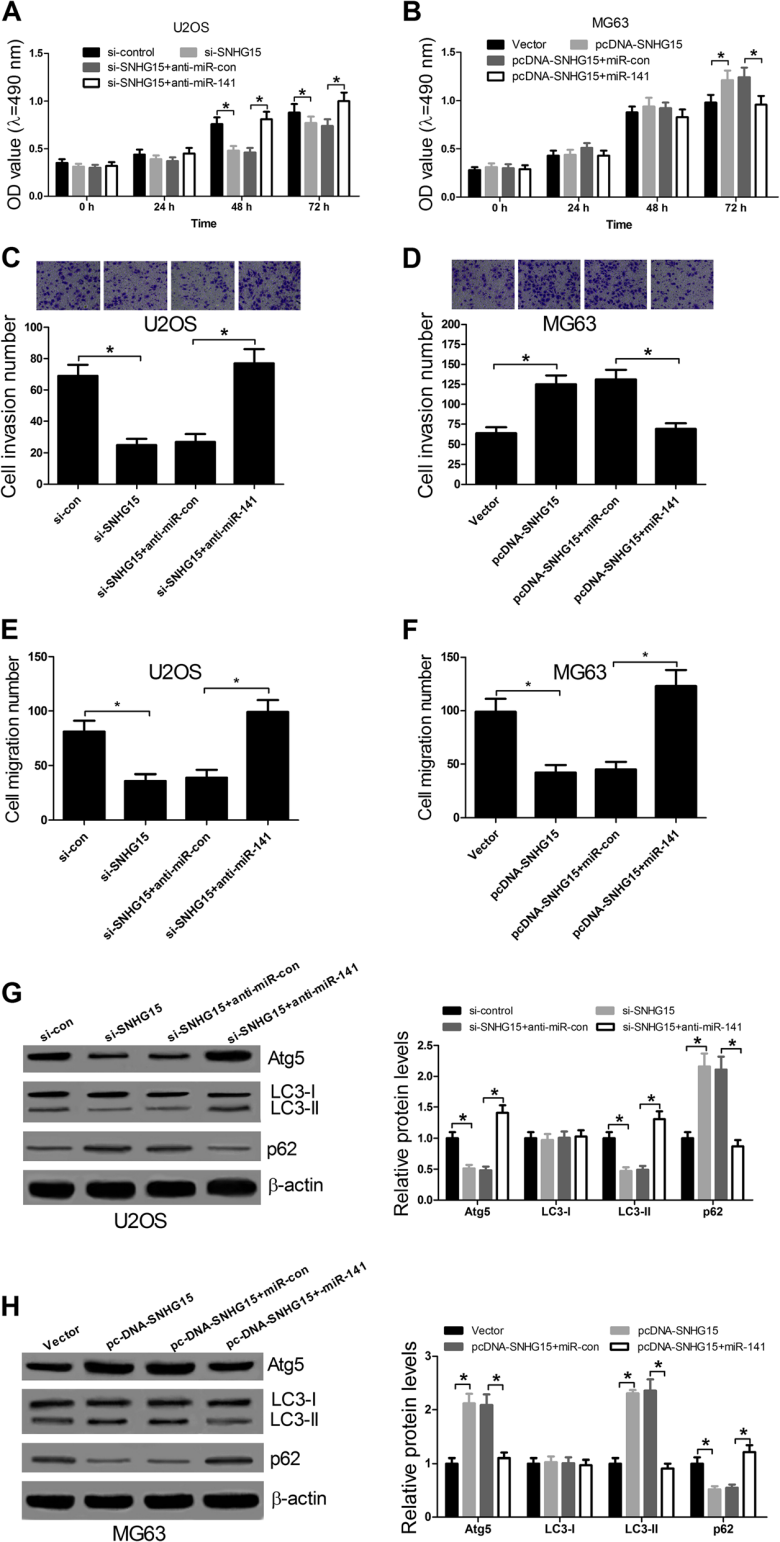
SNHG15 contributed to proliferation, invasion, migration and autophagy by sponging miR-141 in OS cells. U2OS cells were transfected with si-SNHG15, si-control, si-SNHG15 + anti-miR-141, or si-SNHG15 + anti-miR-control. MG63 cells were transfected with pcDNA-SNHG15, vector, pcDNA-SNHG15 + miR-141, or pcDNA-SNHG15 + miR-control. Transfected U2OS and MG63 cells were maintained for 48 h. MTT assay was performed to detect cell proliferation at 24 h, 48 h and 72 h in transfected U2OS (**a**) and MG63 (**b**) cells. Transwell invasion assay was used to assess cell invasiveness in transfected U2OS (**c**) and MG63 (**d**) cells. Transwell migration assay was used to assess cell migration in transfected U2OS (**e**) and MG63 (**f**) cells. Western blot analysis was carried out to determine the levels of Atg5, LC3-I, LC3-II and p62 in transfected U2OS (**g**) and MG63 (**h**) cells. β-actin was used to normalize the levels of Atg5, LC3-I and LC3-II. *P < 0.05 vs. control group

We further explored the effect of regulatory relationship of SNHG15 and miR-141 on biological functions of OS cells. As displayed in Fig. 5a, SNHG15 knockdown led to a significant decrease of cell proliferation at 48 h and 72 h in U2OS cells, while miR-141 suppressing dramatically recuperated this decrease of cell proliferation. In addition, MTT assay also demonstrated that miR-141 conspicuously inhibited the promotion of cell proliferation at 72 h mediated by SNHG15 in MG63 cells (Fig. 5b). Besides, miR-141 down-regulation significantly reversed the inhibition of cell invasiveness by SNHG15 knockdown in U2OS cells (Fig. 5c) and miR-141 overexpression dramatically abated the promotion of cell invasiveness induced by SNHG15 overexpression in MG63 cells (Fig. 5d). Significantly, miR-141 down-regulation significantly reversed the inhibition of cell migration by SNHG15 knockdown in U2OS cells (Fig. 5e) and miR-141 overexpression dramatically abated the promotion of cell migration induced by SNHG15 overexpression in MG63 cells (Fig. 5f). Furthermore, miR-141 repression markedly relieved the reduction of levels of Atg5, LC3-II and the ratio of LC3-II/ LC3-I and the increment of levels of p62 by SNHG15 knockdown in U2OS cells (Fig. 5g). Conversely, miR-141 strikingly abolished the improvement of levels of Atg5, LC3-II and the ratio of LC3-II/ LC3-I and autophagy-related p62 degradation was blocked in MG63 cells induced by SHNG15 (Fig. 5h). These data revealed that SNHG15 contributed to OS cell proliferation, invasion, migration and autophagy by sponging miR-141.

## Discussion

Recently, increasing evidences have demonstrated that lncRNAs play considerable functional roles in a wide variety of physiological and pathological processes involved in tumorigenesis, invasion and metastasis in human malignant cancers [36]. Changes in the primary structure, secondary structure and expression levels of lncRNAs are closely related to cancer formation and progression [37]. As a newly found lncRNA, SNHG15 was reported to be up-regulated in gastric cancer cells in comparison with normal gastric tissues and positively correlated with invasion depth, advanced tumor node metastasis (TNM) stage, lymph node metastasis and poor overall survival [38]. Besides, knockdown of SNHG15 significantly inhibited cell proliferation and invasion and promoted apoptosis, whereas forced expression of SNHG15 exhibited the opposite effects on gastric cancer cells via regulating MMP2 and MMP9 protein expression [38]. Furthermore, SNHG15 may serve as an efficient prognostic biomarker for hepatocellular carcinoma [39]. In our study, we found that SNHG15 was dramatically highly expressed in OS tissues and cells, suggesting that SNHG15 played a pivotal role in OS progression. The biological functions of SNHG15 in OS cells were further investigated by conducting loss or gain of function of SNHG15 assays on OS cellular processes including cell proliferation, invasion, migration and autophagy. The results showed that SNHG15 knockdown markedly suppressed proliferation, invasion, migration and autophagy, while elevated SNHG15 expression contributed to proliferation, invasion, migration and autophagy, indicating that SNHG15 played an oncogenic role in OS cells.

A growing number of studies have demonstrated that abnormal expression of miR-141 played tumor suppressive roles in many cellular processes during tumor occurrence and progression [40, 41]. For example, Peng et al. reported that miR-141 expressed lower in glioma cells and tissues and functioned as tumor suppressor by targeting TGF-β2 [42]. Yao et al. found that miR-141 expression was significantly lower in hepatocellular carcinoma (HCC) and elevated expression of miR-141 inhibited proliferation, invasion and migration of HCC cells [43]. In addition, it was reported that miR-141 was down-regulated in OS and played an OS-suppressing role [9]. Consistent with previous studies, our studies implied that miR-141 was dramatically down-regulated in OS tissues and cells. Besides, we proved that miR-141 overexpression resulted in a significant inhibition on OS cell proliferation, invasion and autophagy, while miR-141 suppressing showed the opposite effects on OS cells, confirming the tumor suppressive roles in OS cells.

Besides, emerging evidences have proved that lncRNAs can sponge miRNA to completely inhibit miRNA function in OS [44]. For example, lncRNA metastasis-associated lung adenocarcinoma transcript 1 (MALAT1) was highly expressed in OS tissues and cells and promoted OS cell growth and tumor progression by inhibiting miR-376a [45]. Besides, tumor-suppressor miR-141 overexpression suppressed osteoblastic cell proliferation by down-regulating lncRNA H19 in OS [12]. In our study, we first disclosed that SNHG15 was inversely correlated with miR-141 expression. Notably, bioinformatics analysis and luciferase reporter system revealed that miR-141 could directly bind to SNHG15. Our study further revealed that SNHG15 negatively regulated miR-141 expression. Meanwhile, rescue experiments showed that repression of miR-141 completely reversed the inhibition of OS cell proliferation, invasion, migration and autophagy by SNHG15 knockdown. In contrast, miR-141 overexpression significantly alleviated SNHG15 overexpression-induced OS cell proliferation, invasion, migration and autophagy. Taken together, these findings suggested that SNHG15 directly interacted with miR-141 and exerted its oncogenic roles by negatively regulating miR-141.

## Conclusions

In conclusion, our study first found that SNHG15 was up-regulated in OS tissues and cells and functioned as an oncogene in regulating OS malignancy by directing interacting with miR-141 and negatively regulating its expression. These results showed that SNHG15 may be a new potential target and prognostic biomarker for the treatment of OS.
